# Low Serum Testosterone Concentrations Are Associated With Poor Cognitive Performance in Older Men but Not Women

**DOI:** 10.3389/fnagi.2021.712237

**Published:** 2021-11-01

**Authors:** Xue Dong, Hong Jiang, Suyun Li, Dongfeng Zhang

**Affiliations:** ^1^Department of Epidemiology and Health Statistics, The School of Public Health, Qingdao University, Qingdao, China; ^2^Shandong Provincial Key Laboratory of Pathogenesis and Prevention of Neurological Disorders and State Key Disciplines: Physiology, Department of Physiology, School of Basic Medicine, Qingdao University, Qingdao, China

**Keywords:** testosterone, cognitive performance, aging, dose-response, NHANES

## Abstract

**Objective:** Current evidence on the association between serum testosterone and cognitive performance has been inconsistent, especially in older adults. To investigate the associations between serum testosterone and cognitive performance in a nationally representative sample of older men and women.

**Methods:** We used data from the National Health and Nutrition Examination Survey (NHANES) 2011–2014. 1,303 men and 1,349 women aged 60 years or older were included in the study. Serum total testosterone was preformed via isotope dilution liquid chromatography tandem mass spectrometry (ID-LC-MS/MS) method. Free testosterone was calculated by Vermeulen’s formula. Cognitive performance was evaluated by the Consortium to Establish a Registry for Alzheimer’s Disease (CERAD) test, Animal Fluency test, and Digit Symbol Substitution Test (DSST). Binary logistic regression and restricted cubic spline models were applied to evaluate the association of testosterone and cognitive performance.

**Results:** In men, higher concentrations of total testosterone were associated with better performance on CERAD test (OR = 0.51; 95%CI = 0.27–0.95) and DSST (OR = 0.54; 95%CI = 0.30–0.99) in adjusted group. Similarly, higher concentrations of free testosterone were associated with better performance on CERAD test (OR = 0.32; 95%CI = 0.17–0.61) and DSST (OR = 0.41; 95%CI = 0.17–0.96) in men. These associations were not seen in women.

**Conclusion:** Serum testosterone concentrations were inversely associated with cognitive performance in older men but not women in the United States.

## Introduction

Cognitive performance, including memory, attention, language and visuospatial ability, declines with age (Harada et al., 2013; Lehert et al., 2015). As life expectancy increases, age-related cognitive decline may be a great health challenge for older adults, cognitive health has become a major public health issue for the aging population in the United States (The Public Health Road Map for State and National Partnerships, 2013–2018). The process from cognitive decline to dementia is continuous and irreversible. In America, approximately 5.1 million people have dementia, and that is expected to double by 2050 (Hebert et al., 2013). The financial burden of dementia has exceeded the costs of cardiovascular and cancer diseases (Ernst and Hay, 1997). The global cost of dementia was $957.56 billion in 2015 and is projected to reach $9.12 trillion by 2050 (Jia et al., 2018). Therefore, the identification of high-risk factors of cognitive decline at early stages may be the most effective strategy for the prevention and therapy of dementia.

Almost all reproductive hormones are secreted by the hypothalamus pituitary gonad axis (Vadakkadath Meethal and Atwood, 2005), such like gonadotropin releasing hormone (GnRH), follicle-stimulating hormone (FSH), luteinizing hormone (LH), and sex hormone. With the aging of the body, gonadal function decreases gradually. This leads to a reduced and chaotic secretion of androgens and estrogens, and an increase secretion of LH and FSH (Gurvich et al., 2018). Previous studies have suggested that an age associated increase in levels of LH is associated with an increased risk of Alzheimer’s disease (Blair et al., 2015), and FSH may exert contrasting effects (Rodrigues et al., 2008). Androgens play a crucial role in human reproductive and sexual function (Hsu et al., 2015a; Davis et al., 2016). In men, Leydig cells of testes are the main sites of androgens production and release. In women, adrenal glands and ovaries represent the main sources of androgens, but for premenopausal women and those over 60, the ovaries produce little testosterone (Davison et al., 2005; Arlt, 2006). Androgens are also needed for muscle formation, body composition and fat metabolism (Orwoll, 2001; Tchernof et al., 2018). Testosterone is the principal androgens. Some studies have pointed out that testosterone could help protect the brain against accelerated cognitive decline due to its neuroprotective effects against oxidative stress (Ahlbom et al., 2001) and apoptosis (Holland et al., 2011). Low levels of testosterone or inadequate use of androgen receptors may impair human development and reproduction (Dohle et al., 2003), and contribute to selective losses in memory and cognitive function (Hamson et al., 2016).

Although a number of epidemiological studies have examined the association between serum testosterone and cognitive performance so far (Fukai et al., 2009; Boss et al., 2014; Hsu et al., 2015b; Giagulli et al., 2016), conclusive conclusions cannot be drawn, in particular, for cognitive performance in older adults. Some cross-sectional and longitudinal studies have indicated that lower testosterone levels may be associated with poorer cognitive performance in older men and women (Wolf and Kirschbaum, 2002; Hogervorst et al., 2010; Hsu et al., 2015b; Giagulli et al., 2016), while other studies have indicated that there was no significant association between serum testosterone and cognitive performance (Koyama et al., 2016; Zhao et al., 2016; Kische et al., 2017). These inconsistent results prompted us to elucidate the precise role of testosterone in cognitive performance in older adults.

Therefore, we analyzed a nationally representative sample of older men and women aged 60 years or older in the National Health and Nutrition Examination Survey (NHANES) to investigate the associations between serum testosterone and cognitive performance.

## Materials and Methods

### Data Collection and Study Population

The NHANES is a 2-year-cycle cross-sectional survey conducted by the Centers for Disease Control and Prevention (CDC) of America (‘NHANES. National Center for Health Statistics’). Representative of the civilian, non-institutionalized United States population were selected by a complex, multistage, probability sampling design. The NHANES protocols were approved by the National Center for Health Statistics Ethics Review Board of the United States CDC, and all participants signed informed consent during the survey.

**FIGURE 1:**
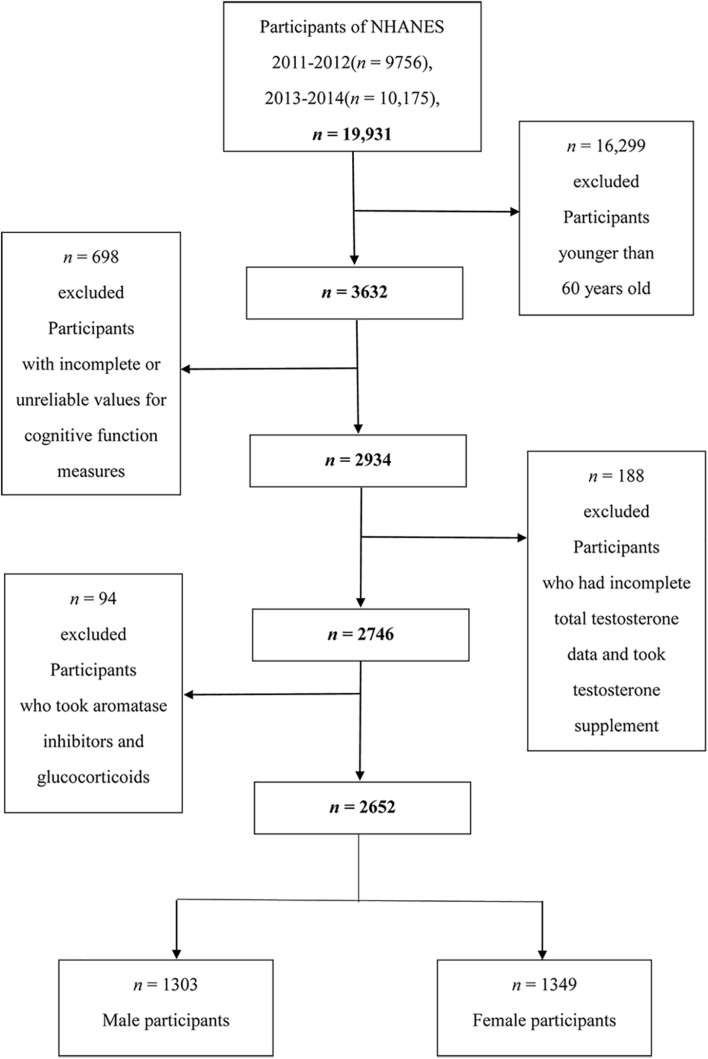
Flow chart of the screening process for the selection of eligible participants.

Two cycles (2011–2012 and 2013–2014) were combined and used in the analysis. The data needed for these analyses were not available in NHANES surveys 2015–2020, so we did not include these cycles. A total of 19,931 individuals were included in the study, and our analyses were limited to 3,632 individuals aged 60 years or older. Among them, we excluded participants with incomplete cognitive tests or with unreliable values for the three cognition tests (*n* = 698), and we further excluded participants who had incomplete total testosterone data and took testosterone supplement (*n* = 188). We then excluded participants who took aromatase inhibitors and glucocorticoids (*n* = 94). After exclusions, a total of 2,652 participants aged 60 years or older were included in this study (1,303 men and 1,349 women) (Figure 1).

### Cognitive Performance Assessment

In 2011–2014, a series of assessments for cognitive performance in NHANES were introduced among participants aged 60 years or older (‘NHANES 2011–2012 Data Documentation, Cognitive Functioning’), including: the Consortium to Establish a Registry for Alzheimer’s Disease (CERAD) Word Learning sub-test; the Animal Fluency test; and the Digit Symbol Substitution Test (DSST). Although cognitive assessments cannot replace diagnoses based on clinical examinations, they have been used in large-scale screening and epidemiological studies to examine the associations between cognitive performance and many of the medical conditions and risk factors measured in NHANES examination (Fillenbaum et al., 2008; Gao et al., 2009; Clark et al., 2009; Jaeger, 2018). The scoring rules of three cognitive tests in this study are consistent with our previous studies (Dong et al., 2020a).

Currently, there is no gold standard of cutoff point for the CERAD, Animal Fluency, and DSST test to identify low cognitive performance. Same as the methods used in the published literature (Chen et al., 2017; Li et al., 2019), we used the 25th percentile of the score, the lowest quartile, as the cutoff point. Considering the significant effect of age on cognitive performance, the score was further categorized based on age (60–<70 years, 70–<80 years, and ≥80 years). For each dimension, participants were divided into two groups: the low cognitive performance group and the normal cognitive performance group.

### Serum Testosterone Assessment

All participants had their blood drawn (venipuncture) in the morning session after an overnight fast. Serum total testosterone was preformed via isotope dilution liquid chromatography tandem mass spectrometry (ID-LC-MS/MS) method for routine quantitation of serum total testosterone based on the National Institute for Standards and Technology’s (NIST) reference method (‘NHANES 2013–2014, Sex Steroid Hormone’).

Free testosterone was calculated from total testosterone, sex hormone-binding globulin (SHBG) and albumin (already available in the NHANES 2013–2014) using a validated equation derived from the mass action law as described by Vermeulen et al. (1999) and Woodward et al. (2020). SHBG is the blood transport protein for androgens and estrogens, it was measured via the reaction of SHBG with immuno-antibodies and chemo-luminescence measurements of the reaction products. The DcX800 method was used to measure the albumin concentration as a bichromatic digital endpoint method. Serum total and calculated free testosterone were categorized into quartiles.

### Covariates

In addition to serum testosterone, we investigated some potential confounding factors, which included: age, race, living arrangements, educational level, body mass index (BMI), drinking, smoking, charlson comorbidity index (CCI), work and recreational activity, employment status, poverty–income ratio, depressive symptoms, hemoglobin, hysterectomy, oophorectomy and the age of menopause for women. The classifications of covariates were based on our previous studies (Dong et al., 2020a, b) and are shown in Table 1.

**TABLE 1:** Characteristics of the study population disaggregated by quartiles of total testosterone, NHANES 2011–2014 (*N* = 2,652).

	Testosterone quartiles (nmol/L)							
	Men				Women			
	<9.3	9.3–<12.6	12.6–<17.4	>17.4	<0.3	0.3–<0.56	0.56–<0.85	>0.85
Number of subjects (%)	326(25)	326(25)	326(25)	325(25)	338(25)	337(25)	338(25)	336(25)
**Age (%)**								
60–70 years	159(48.8)	177(54.3)	172(52.8)	214(65.8)	183(54.1)	188(55.8)	197(58.3)	161(47.9)
70–80 years	93(28.5)	102(31.3)	103(31.6)	70(21.6)	101(29.9)	94(27.9)	94(27.8)	112(33.3)
≥80 years	74(22.7)	47(14.4)	51(15.6)	41(12.6)	54(16.0)	55(16.3)	47(13.9)	63(18.8)
**Race (%)**								
Mexican American	36(11.0)	28(8.6)	32(9.8)	31(9.5)	35(10.4)	3911.6)	24(7.1)	16(4.8)
Other Hispanic	26(8.0)	33(10.1)	30(9.2)	40(12.3)	33(9.8)	36(10.7)	45(13.3)	29(8.6)
Non-Hispanic white	164(50.3)	146(44.8)	154(47.2)	139(42.8)	170(50.3)	173(51.3)	159(47.0)	177(52.7)
Non-Hispanic black	73(22.4)	82(25.2)	74(22.7)	82(25.2)	66(19.5)	57(16.9)	78(23.1)	91(27.1)
Other race	27(8.3)	37(11.3)	36(11.1)	33(10.2)	34(10.1)	32(9.5)	32(9.5)	23(6.8)
**Educational level (%)**								
Below high school	87(26.7)	79(24.3)	83(25.5)	82(25.2)	81(24.0)	86(25.5)	103(30.5)	63(18.8)
High school	79(24.2)	80(24.6)	62(19.1)	79(24.3)	87(25.7)	92(27.3)	65(19.2)	89(26.5)
Above high school	160(49.1)	166(51.1)	180(55.4)	164(50.5)	170(50.3)	159(47.2)	170(50.3)	184(54.8)
**Living arrangements (%)**								
Living with other adults	276(84.7)	271(83.1)	255(78.2)	250(76.9)	238(70.4)	228(67.7)	239(70.7)	221(65.8)
Living alone	50(15.3)	55(16.9)	71(21.8)	75(23.1)	100(29.6)	109(32.3)	99(29.3)	115(34.2)
**Poverty–income ratio (%)**								
≤0.99	59(19.2)	74(23.6)	74(24.0)	63(20.3)	86(26.6)	88(26.9)	96(29.9)	97(29.8)
≥1	249(80.8)	240(76.4)	234(76.0)	248(79.7)	237(73.4)	239(73.1)	225(70.1)	228(70.2)
**Body mass index (%)**								
<25 kg/m^2^	51(15.9)	46(14.4)	95(29.7)	140(43.5)	99(29.5)	102(30.8)	89(26.6)	80(24.2)
25–30 kg/m^2^	113(35.2)	135(42.2)	146(45.6)	136(42.2)	120(35.7)	97(29.3)	97(29.0)	91(27.6)
≥30 kg/m^2^	157(48.9)	139(43.4)	79(24.7)	46(14.3)	117(34.8)	132(39.9)	149(44.5)	159(48.2)
**Work activity (%)**								
Vigorous	41(12.6)	53(16.3)	57(17.5)	59(18.2)	21(6.2)	18(5.3)	20(5.9)	21(6.3)
Moderate	70(21.5)	61(18.8)	60(18.4)	57(17.6)	70(20.7)	72(21.4)	63(18.6)	74(22.0)
Other	214(65.8)	211(64.9)	209(64.1)	208(64.2)	247(73.1)	247(73.3)	255(75.4)	241(71.7)
**Recreational activity (%)**								
Vigorous	30(9.2)	27(8.3)	36(11.0)	48(14.8)	35(10.4)	20(5.9)	25(7.4)	28(8.3)
Moderate	100(30.7)	117(35.9)	125(38.3)	92(28.3)	103(30.5)	123(36.5)	107(31.7)	106(31.5)
Other	196(60.1)	182(55.8)	165(50.7)	185(56.9)	200(59.2)	194(57.6)	206(60.9)	202(60.2)
Had at least 12 alcohol drinks/year (%)	264(82.5)	268(84.0)	270(84.6)	267(83.7)	166(49.7)	190(57.2)	183(55.3)	182(54.8)
Smoke at least 100 cigarettes in life (%)	202(62.0)	221(67.8)	184(56.4)	216(66.7)	124(36.7)	117(34.8)	128(37.9)	142(42.3)
**Employment status (%)**								
Unemployed	239(73.3)	224(68.7)	220(67.5)	212(65.4)	257(76.0)	253(75.3)	256(75.7)	257(76.5)
Employed	87(26.7)	102(31.3)	106(32.5)	112(34.6)	81(24.0)	83(24.7)	82(24.3)	79(23.5)
Depressive symptoms (%)	27(8.5)	21(6.6)	21(6.6)	20(6.3)	34(10.2)	34(10.2)	22(6.6)	12(3.6)
Hemoglobin (g/dL), median (interquartile range)	14.1(2.2)	14.3(1.8)	14.7(1.7)	14.7(1.8)	13.1(1.5)	13.3(1.5)	13.3(1.5)	13.5(1.5)
Charlson comorbidity index, median (interquartile range)	3.0(2.0)	2.0(3.0)	2.0(2.0)	2.0(2.0)	2.0(3.0)	2.0(2.0)	2.0(2.0)	2.0(2.0)
**CERAD test (%)**								
Normal cognitive performance	206(63.2)	240(73.6)	233(70.5)	229(70.5)	256(75.7)	251(74.5)	253(74.9)	233(69.3)
Low cognitive performance	120(36.8)	86(26.4)	93(29.5)	96(29.5)	82(24.3)	86(25.5)	85(25.1)	103(30.7)
**Animal fluency test (%)**								
Normal cognitive performance	219(67.2)	231(70.9)	243(74.5)	236(72.6)	234(69.2)	240(71.2)	237(70.1)	234(69.6)
Low cognitive performance	107(32.8)	95(29.1)	83(25.5)	89(27.4)	104(30.8)	97(28.8)	101(29.9)	102(30.4)
**DSST (%)**								
Normal cognitive performance	235(72.1)	245(75.2)	256(78.5)	236(72.6)	253(74.9)	250(74.2)	256(75.7)	239(71.1)
Low cognitive performance	91(27.9)	81(24.8)	70(21.5)	89(27.4)	85(25.1)	87(25.8)	82(24.3)	97(28.9)

*Data is number of subjects (percentage) or medians (interquartile ranges).*

The CCI, calculated from Charlson et al. (1987), is a commonly used index for assessing comorbidities and indicating overall health. The CCI contains 19 chronic disease items (Supplementary Table 1). As NHANES did not collect data on some components of CCI (e.g., dementia, hemiplegia, HIV/AIDS, and metastatic solid tumor), these components were excluded from CCI used in the current study. Scores for each disease item were summed to generate a total CCI score that could range from 0 to 10 points, with higher scores indicating greater comorbidities.

### Statistical Analysis

Statistical analyses were performed by Stata 15.0 (Stata Corporation, College Station, TX, United States). A new sample weight was constructed according to the analytical guidelines of the NHANES (‘NHANES. Tutorials. Module 3: Weighting’). The Kolmogorov-Smirnov normality test was adopted to test the normality of continuous variables. Normally distributed variables were described with mean ± standard deviation, non-normally distributed variables were described with median (interquartile range). We conducted binary logistic regression analyses to examine the associations of serum total and free testosterone with cognitive performance in older men and women. Regression equations (‘NHANES 2013–2014, Sex Steroid Hormone’) were used when combining data of total testosterone across 2011–2012 and 2013–2014 cycles. Based on prior studies and theoretical considerations (Fukai et al., 2009; Hsu et al., 2015b; Christensen et al., 2018; Li et al., 2019), we selected established risk factors for cognitive performance that were also known to be associated with testosterone. Crude Model did not adjust any confounders. Model 1 adjusted for age. Model 2 additionally adjusted for race, educational level, living arrangements, employment status, CCI, BMI, drinking status, smoking status, work activity, recreational activity, income, depressive symptoms, hemoglobin, hysterectomy, oophorectomy and the age of menopause for women. We further used restricted cubic spline with three knots located at the 5th, 50th, and 95th percentiles of the exposure distribution to assess the dose–response relationship in Model 2. A two-sided *p* < 0.05 was considered statistically significant.

## Results

A total of 2,652 participants aged 60 years or older were included in this study (49.1% were men and 50.9% were women). Detailed characteristics of the study population were shown in Table 1. Total testosterone levels by 5-year age groups in men and in women were provided in Supplementary Table 2.

Table 2 shows the associations between serum total testosterone and different dimensions of cognitive performance in men in NHANES 2011–2014. The crude odds ratio (OR) with 95% confidence interval (CI) of CERAD test (verbal memory) was 0.48 (0.30–0.77) for the highest versus the lowest quartile of total testosterone. After adjustment for age, serum testosterone was still associated with cognitive performance. In Model 2, the multivariate-adjusted OR (95% CI) of CERAD test (verbal memory) was 0.51 (0.27–0.95) for the highest versus the lowest quartile of total testosterone. The crude OR (95% CI) of DSST (processing speed) was 0.58 (0.37–0.90) for the third versus the lowest quartile of total testosterone. After adjustment for age, serum testosterone was still associated with cognitive performance. In Model 2, the multivariate-adjusted OR (95% CI) of DSST (processing speed) was 0.54 (0.30–0.99) for the third versus the lowest quartile of total testosterone. The association between serum total testosterone and Animal Fluency test was not statistically significant.

**TABLE 2:** Weighted odds ratios (95% confidence intervals) for score on CERAD, animal fluency and DSST test across quartiles of total testosterone in men, NHANES 2011–2014 (*N* = 1,303).

	Quartile of total testosterone			
	Q1	Q2	Q3	Q4
**Total testosterone (nmol/L)**	**<9.3**	**9.3–<12.6**	**12.6–<17.4**	**>17.4**
**CERAD test**				
Crude^a^	1.00 (Ref.)	0.59 (0.36–0.97)*	0.68 (0.47–1.00)	0.48 (0.30–0.77)**
Model 1^a^	1.00 (Ref.)	0.62 (0.37–1.03)	0.73 (0.49–1.11)	0.59 (0.35–0.99)*
Model 2^a^	1.00 (Ref.)	0.63 (0.33–1.18)	0.69 (0.42–1.13)	0.51 (0.27–0.95)*
**Animal fluency test**				
Crude^a^	1.00 (Ref.)	0.78 (0.48–1.28)	0.64 (0.37–1.09)	0.87 (0.45–1.72)
Model 1^a^	1.00 (Ref.)	0.84 (0.54–1.34)	0.69 (0.40–1.19)	1.05 (0.54–2.06)
Model 2^a^	1.00 (Ref.)	0.82 (0.53–1.28)	0.67 (0.37–1.19)	1.03 (0.52–2.05)
**DSST**				
Crude^a^	1.00 (Ref.)	0.74 (0.48–1.15)	0.58 (0.37–0.90)*	0.80 (0.45–1.43)
Model 1^a^	1.00 (Ref.)	0.81 (0.55–1.18)	0.63 (0.42–0.96)*	0.99 (0.56–1.77)
Model 2^a^	1.00 (Ref.)	0.68 (0.42–1.10)	0.54 (0.30–0.99)*	1.03 (0.42–2.47)

*^a^Calculated using binary logistic regression.*

*Model 1 adjusted for age.*

*Model 2 adjusted for age, race, educational level, living arrangements, employment status, CCI, BMI, drinking status, smoking status, work activity, recreational activity, income, depressive symptoms, and hemoglobin.*

*p < 0.05, **p < 0.01.

Supplementary Table 3 shows the associations between serum free testosterone and different dimensions of cognitive performance in men in NHANES 2013–2014. The crude OR (95% CI) of CERAD test (verbal memory) was 0.26 (0.13–0.53) for the third versus the lowest quartile of free testosterone. After adjustment for age, serum testosterone was still associated with cognitive performance. In Model 2, the multivariate-adjusted OR (95% CI) of CERAD test (verbal memory) was 0.32 (0.17–0.61) for the third versus the lowest quartile of free testosterone. The crude OR (95% CI) of DSST (processing speed) was 0.38 (0.16–0.89) for the third versus the lowest quartile of free testosterone. After adjustment for age, serum testosterone was still associated with cognitive performance. In Model 2, the multivariate-adjusted OR (95% CI) of DSST (processing speed) was 0.41 (0.17–0.96) for the third versus the lowest quartile of free testosterone. The association between free testosterone and Animal Fluency test was not statistically significant. The associations between serum total and free testosterone and different dimensions of cognitive performance in women are shown in Supplementary Tables 4, 5. No significant associations were observed between total and free testosterone and cognitive performance in three tests.

**FIGURE 2:**
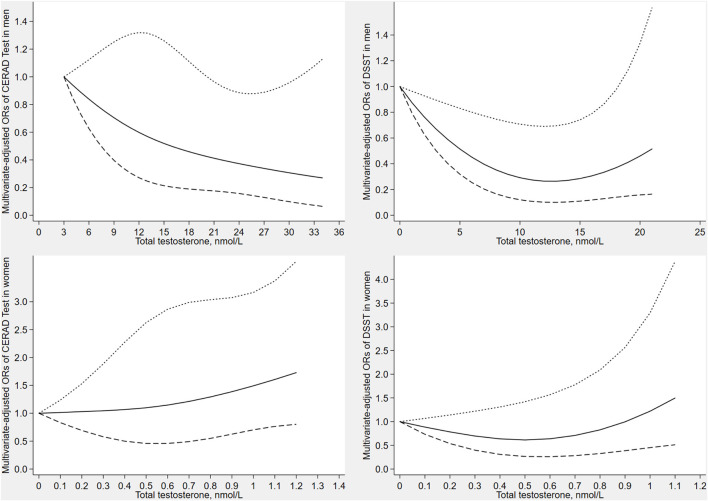
Dose–response relationship between serum total testosterone with CERAD test score and DSST score in men and women. The solid line represents the estimated odds ratio and the area bound by the dashed lines represent the 95% confidence interval.

Figure 2 depicts the results of the restricted cubic spline analyses in men and women. The prevalence of low cognitive performance decreased with increasing levels of testosterone in CERAD test (verbal memory), and showed a non-linear dose-response relationship (*p* for non-linearity = 0.041). Similarly, the prevalence of low cognitive performance decreased with increasing levels of testosterone in DSST (processing speed), and showed a non-linear dose-response relationship (*p* for non-linearity = 0.037). No significant associations were observed between total testosterone and cognitive performance in women.

## Discussion

In this study, we combined data from NHANES 2011–2012 and 2013–2014 and included 1,303 men and 1,349 women aged 60 years or older. In men, in model 2, the associations between serum total testosterone and CERAD test (verbal memory) and DSST (processing speed) were significant. No significant association was observed between serum total testosterone and Animal Fluency test (verbal fluency). We further examined the associations between serum free testosterone and cognitive performance in NHANES 2013–2014. We found that free testosterone was associated with CERAD test (verbal memory) and DSST (processing speed) in men. These associations were not seen in women.

Our finding about serum testosterone in men was in part consistent with those of some previous studies (Matousek and Sherwin, 2010; Hsu et al., 2015b; Koyama et al., 2016; Tan et al., 2019). A population-based study conducted by Hsu et al. (2015b) showed that higher testosterone levels predicted better performance on several tests of cognitive function in older men. Hogervorst et al. (2010) found that higher testosterone was associated with better cognition in older men both at baseline and the 2-year follow-up. Moreover, a cross-sectional study (Fukai et al., 2009) of 208 older people (108 men and 100 women) suggested that testosterone was related to cognitive function in older men. Whereas some studies (Fonda et al., 2005; Lee et al., 2010; Ryan et al., 2012; Zhao et al., 2016) showed inconsistent results. A large prospective cohort study (Zhao et al., 2016) of 4,212 older men did not corroborate observed protective effect of testosterone on cognitive performance. One community-based, cross-sectional study (Lee et al., 2010) of 3,369 men aged 40–79 years suggested that no significant association was observed of testosterone with cognition after multivariable adjustment.

Some epidemiological studies investigating the role of testosterone in women were consistent with present study. A 23-year prospective cohort study of 3,044 women (Koyama et al., 2016) indicated that no significant association was found between total testosterone levels and cognitive performance in later life. Longitudinal data from 4,110 participants of the Study (Kische et al., 2017) indicated that serum testosterone levels were not associated with cognitive function in older women. Moreover, a cross-sectional study (Fukai et al., 2009) of 208 older people found no significant association between endogenous testosterone and cognition in older women. Nevertheless, some studies showed inconsistent results (Drake et al., 2000; Wolf and Kirschbaum, 2002). A community-based, cross-sectional study (Wolf and Kirschbaum, 2002) of 38 postmenopausal women suggested that higher testosterone levels were associated with better cognitive performance. In a longitudinal study of aging and dementia, Drake et al. (2000) found that higher testosterone levels were associated with better cognition in older women. Besides, several randomized placebo-controlled trials (Shah et al., 2006; Kocoska-Maras et al., 2011; Davis et al., 2014) investigated the association between testosterone therapy and cognition in postmenopausal women, some of which had shown benefits (Shah et al., 2006; Davis et al., 2014). The different conclusions could possibly arise from different study design, studied samples or differences in the tests used to assess cognitive function. Further studies are warranted to better understand the inconsistency of the existing literature on testosterone and cognition in the elderly.

There have been several possible mechanisms to explain the association between testosterone and cognitive performance. The hippocampus is the basis of spatial abilities and declarative memory and contains androgen and estrogen receptors. In the brain, testosterone may have direct effects on the hippocampus through androgen receptors and indirect effects from aromatization to estradiol interacting through estrogen receptors (Oberlander et al., 2004). Cherrier et al. (2005) found that the improvement in verbal memory induced by testosterone in older men depends on the aromatization of testosterone to estradiol, but the effect of testosterone to improve spatial memory depends on the direct action of testosterone to androgen receptors. Furthermore, testosterone has been shown to increase concentrations of nerve growth factor (NGF) in the hippocampus and upregulate the NGF receptor in the forebrain (Tirassa et al., 1997). Androgens can also inhibit the excitotoxicity of *N*-methyl-D-aspartate receptor in hippocampal neurons and promote the growth and sprouting of nerve fibers (Pouliot et al., 1996). In addition, testosterone can improve synaptic plasticity through the mediation of androgen receptor (Jia et al., 2016). Patients with Alzheimer’s disease (AD) are characterized by an early impairment of the mechanisms of synaptic plasticity (Di Lorenzo et al., 2020) that is associated with neuropsychological deficits (Di Lorenzo et al., 2019).

The explanations for the sex differences in the association between testosterone and cognitive performance could be due to sex differences in hormone secretion and metabolism (Juster et al., 2016). Serum testosterone levels in women were much lower than in men, providing a possible explanation for no association between testosterone levels and cognition in women. Moreover, Bezdickova et al. (2007) reported that nuclear androgen receptor staining was observed in the mammillary body, precentral gyrus and hippocampus in the male brain but not in the female brain. The sex difference in the association between testosterone and cognitive performance may be further determined based on the ligand–receptor relationship.

Our study presents several advantages. First, we used a large nationally representative sample of older adults in the United States. Second, we controlled some potential confounders to provide a better estimate of associations between testosterone and cognitive performance. Third, we investigated the dose–response relationship between testosterone and cognitive performance.

Our study has several limitations. Primarily, we were unable to perform a longitudinal study due to the limited data, so it was difficult to generalize the results of this study from a cause-effect relationship between serum testosterone levels and cognitive performance. Furthermore, the cognitive tests did not cover all domains of cognitive function, adults who performed well in one domain may not perform well in another domain. Moreover, we only analyzed the association between free testosterone and cognitive performance in one cycle because of the finiteness of measured data, and free testosterone was calculated using a validated equation derived from the mass action law rather than directly measured via ID-LC-MS/MS method, which may not accurately reflect individuals’ level. Besides, it was hard to differentiate moderate-heavy alcohol use from little-no use, which might affect results. In addition, the data for estrone and adrenal androgen precursors (DHEAS and androstenedione) which have been shown to correlate with cognitive performance in older women were not available in NHANES surveys 2011–2014. Finally, although we excluded participants who took aromatase inhibitors and glucocorticoids, there were still multiple medications that would need excluding, which might affect testosterone levels.

Our study suggested that serum total and free testosterone were inversely associated with low cognitive performance for older men aged 60 years or older in the United States. No significant association was observed between serum testosterone and cognitive performance in older women. This study might help to understand the possible mechanisms between testosterone and cognitive performance, and provide strategies for the prevention and treatment of cognitive decline. Clinical placebo-controlled trials are needed to determine whether testosterone supplementation in older adults with low testosterone levels may reduce the risk of cognitive decline.

## Data Availability Statement

The original contributions presented in the study are included in the article/Supplementary Material, further inquiries can be directed to the corresponding authors.

## Author Contributions

XD, DZ, and HJ conceived and designed the study. XD, HJ, and SL analyzed the data. XD wrote the manuscript. DZ and HJ reviewed the manuscript and had primary responsibility for the final content. All authors provided critical revisions of the manuscript and approved the final manuscript.

## Conflict of Interest

The authors declare that the research was conducted in the absence of any commercial or financial relationships that could be construed as a potential conflict of interest.

## Publisher’s Note

All claims expressed in this article are solely those of the authors and do not necessarily represent those of their affiliated organizations, or those of the publisher, the editors and the reviewers. Any product that may be evaluated in this article, or claim that may be made by its manufacturer, is not guaranteed or endorsed by the publisher.
