# Supplementary material for: Low Serum Testosterone Concentrations Are Associated With Poor Cognitive Performance in Older Men but Not Women
Source: Front Aging Neurosci. 2021 Nov 1;13:712237. doi: 10.3389/fnagi.2021.712237 (PMC8591394; doi:10.3389/fnagi.2021.712237)
Supplement: Supplementary file 1 [file Table_1.DOCX]

Supplementary Material

| **Supplementary Table 1.** Weighted index of Charlson comorbidity. | |
| --- | --- |
| Charlson comorbidity | Assigned weights for diseases |
| Myocardial infarct | 1 |
| Congestive heart failure | 1 |
| Peripheral vascular disease | 1 |
| Cerebrovascular disease | 1 |
| Dementia | 1 |
| Chronic pulmonary disease | 1 |
| Connective tissue disease | 1 |
| Ulcer disease | 1 |
| Mild liver disease | 1 |
| Diabetes | 1 |
| Hemiplegia | 2 |
| Moderate or severe renal disease | 2 |
| Diabetes with end organ damage | 2 |
| Any tumor | 2 |
| Leukemia | 2 |
| Lymphoma | 2 |
| Moderate or severe liver disease | 3 |
| Metastatic solid tumor | 6 |
| AIDS | 6 |

| **Supplementary Table 2.** Total testosterone levels by 5-year age groups in men and women, NHANES 2011-2014 (N =2,652). | | | | | |
| --- | --- | --- | --- | --- | --- |
|  | Age group | | | | |
|  | 60 to <65Years | 65 to <70Years | 70 to <75Years | 75 to <80Years | ≥80 Years |
| Men | | | | | |
| Total testosterone (nmol/L) | 13.1(9.7,18.0) | 13.1(9.7,18.0) | 12.3(9.9,16.3) | 12.1(8.5,16.1) | 11.4(7.6,15.6) |
| Women | | | | | |
| Total testosterone (nmol/L) | 0.56(0.37,0.83) | 0.54(0.35,0.78) | 0.53(0.37,0.92) | 0.59(0.36,0.96) | 0.56(0.35,0.96) |
| Data is presented with medians (interquartile ranges). | | | | | |

| **Supplementary Table 3.** Weighted odds ratios (95% confidence intervals) for score on CERAD, Animal Fluency and DSST test across quartiles of free testosterone in men, NHANES 2013-2014 (N =616). | | | | |
| --- | --- | --- | --- | --- |
|  | Quartile of free testosterone | | | |
|  | Q1 | Q2 | Q3 | Q4 |
| Free testosterone (nmol/L) | <0.15 | 0.15 to <0.20 | 0.20 to <0.25 | >0.25 |
| CERAD test |  |  |  |  |
| Crude ^a^ | 1.00 (Ref.) | 0.70 (0.36–1.35) | 0.26 (0.13–0.53) ** | 0.48 (0.23–0.99) * |
| Model 1 ^a^ | 1.00 (Ref.) | 0.97 (0.48–1.95) | 0.40 (0.22–0.75) ** | 0.86 (0.44–1.67) |
| Model 2 ^a^ | 1.00 (Ref.) | 0.97 (0.44–2.16) | 0.32 (0.17–0.61) ** | 0.83 (0.46–1.49) |
| Animal Fluency test |  |  |  |  |
| Crude ^a^ | 1.00 (Ref.) | 0.85 (0.40–1.81) | 0.74 (0.30–1.83) | 0.44 (0.23–0.83) * |
| Model 1 ^a^ | 1.00 (Ref.) | 1.04 (0.47–2.31) | 0.99 (0.39–2.52) | 0.62 (0.31–1.25) |
| Model 2 ^a^ | 1.00 (Ref.) | 1.08 (0.44–2.67) | 0.85 (0.32–2.24) | 0.47 (0.19–1.14) |
| DSST |  |  |  |  |
| Crude ^a^ | 1.00 (Ref.) | 0.60 (0.29–1.24) | 0.38 (0.16–0.89) * | 0.43 (0.15–1.20) |
| Model 1 ^a^ | 1.00 (Ref.) | 0.74 (0.35–1.53) | 0.49 (0.24–0.99) * | 0.69 (0.28–1.64) |
| Model 2 ^a^ | 1.00 (Ref.) | 0.67 (0.22–2.02) | 0.41 (0.17–0.96) * | 0.68 (0.25–1.85) |
| ^a^ Calculated using binary logistic regression.  Model 1 adjusted for age.  Model 2 adjusted for age, race, educational level, living arrangements, employment status, CCI, BMI, drinking status, smoking status, work activity, recreational activity, income, depressive symptoms, estradiol and hemoglobin.  **p* < 0.05; ***p* < 0.01. | | | | |

| **Supplementary Table 4.** Weighted odds ratios (95% confidence intervals) for score on CERAD, Animal Fluency and DSST test across quartiles of total testosterone in women, NHANES 2011-2014 (N =1,349). | | | | |
| --- | --- | --- | --- | --- |
|  | Quartile of total testosterone | | | |
|  | Q1 | Q2 | Q3 | Q4 |
| Total testosterone (nmol/L) | <0.3 | 0.3 to <0.56 | 0.56 to <0.85 | >0.85 |
| CERAD test |  |  |  |  |
| Crude ^a^ | 1.00 (Ref.) | 1.10 (0.63–1.92) | 1.01 (0.51–1.95) | 1.29 (0.81–2.06) |
| Model 1 ^a^ | 1.00 (Ref.) | 1.15 (0.69–1.91) | 1.06 (0.57–1.99) | 1.27 (0.82–1.99) |
| Model 2 ^a^ | 1.00 (Ref.) | 1.23 (0.68–2.23) | 1.03 (0.47–2.23) | 1.63 (0.89–2.98) |
| Animal Fluency test |  |  |  |  |
| Crude ^a^ | 1.00 (Ref.) | 0.92 (0.58–1.44) | 0.96 (0.63–1.45) | 0.94 (0.59–1.51) |
| Model 1 ^a^ | 1.00 (Ref.) | 0.92 (0.63–1.36) | 0.99 (0.68–1.42) | 0.89 (0.56–1.41) |
| Model 2 ^a^ | 1.00 (Ref.) | 0.99 (0.60–1.67) | 0.85 (0.49–1.49) | 0.90 (0.53–1.65) |
| DSST |  |  |  |  |
| Crude ^a^ | 1.00 (Ref.) | 0.81 (0.54–1.23) | 0.94 (0.58–1.51) | 1.23 (0.73–2.06) |
| Model 1 ^a^ | 1.00 (Ref.) | 0.82 (0.56–1.21) | 0.99 (0.62–1.60) | 1.20 (0.71–2.03) |
| Model 2 ^a^ | 1.00 (Ref.) | 0.86 (0.48–1.51) | 0.68 (0.34–1.41) | 1.61 (0.79–3.01) |
| ^a^ Calculated using binary logistic regression.  Model 1 adjusted for age.  Model 2 adjusted for age, race, educational level, living arrangements, employment status, CCI, BMI, drinking status, smoking status, work activity, recreational activity, income, depressive symptoms, hemoglobin, hysterectomy, oophorectomy and the age of menopause for women.  **p* < 0.05; ***p* < 0.01. | | | | |

| **Supplementary Table 5.** Weighted odds ratios (95% confidence intervals) for score on CERAD, Animal Fluency and DSST test across quartiles of free testosterone in women, NHANES 2013-2014 (N =670). | | | | |
| --- | --- | --- | --- | --- |
|  | Quartile of free testosterone | | | |
|  | Q1 | Q2 | Q3 | Q4 |
| Free testosterone (nmol/L) | <0.004 | 0.004 to <0.006 | 0.006 to <0.010 | >0.010 |
| CERAD test |  |  |  |  |
| Crude ^a^ | 1.00 (Ref.) | 0.93 (0.42–2.03) | 1.05 (0.60–1.85) | 0.99 (0.42–2.34) |
| Model 1 ^a^ | 1.00 (Ref.) | 1.05 (0.47–2.35) | 1.19 (0.68–2.09) | 1.13 (0.48–2.67) |
| Model 2 ^a^ | 1.00 (Ref.) | 1.16 (0.50–2.74) | 1.21 (0.55–2.65) | 1.21 (0.42–3.47) |
| Animal Fluency test |  |  |  |  |
| Crude ^a^ | 1.00 (Ref.) | 0.93 (0.49–1.73) | 1.01 (0.64–1.59) | 0.80 (0.48–1.36) |
| Model 1 ^a^ | 1.00 (Ref.) | 1.01 (0.51–1.95) | 1.08 (0.66–1.78) | 0.87 (0.50–1.53) |
| Model 2 ^a^ | 1.00 (Ref.) | 1.08 (0.48–2.47) | 0.92 (0.50–1.69) | 0.88 (0.38–2.03) |
| DSST |  |  |  |  |
| Crude ^a^ | 1.00 (Ref.) | 0.80 (0.36–1.76) | 1.17 (0.72–1.90) | 1.15 (0.46–2.83) |
| Model 1 ^a^ | 1.00 (Ref.) | 0.92 (0.41–2.07) | 1.37 (0.78–2.41) | 1.37 (0.55–3.45) |
| Model 2 ^a^ | 1.00 (Ref.) | 1.19 (0.31–3.99) | 1.58 (0.61–4.03) | 2.20 (0.49–4.99) |
| ^a^ Calculated using binary logistic regression.  Model 1 adjusted for age.  Model 2 adjusted for age, race, educational level, living arrangements, employment status, CCI, BMI, drinking status, smoking status, work activity, recreational activity, income, depressive symptoms, hemoglobin, hysterectomy, oophorectomy and the age of menopause for women.  **p* < 0.05; ***p* < 0.01. | | | | |
